# Stability and Plasticity of Contextual Modulation in the Mouse Visual Cortex

**DOI:** 10.1016/j.celrep.2016.12.080

**Published:** 2017-01-24

**Authors:** Adam Ranson

**Affiliations:** 1 Neuroscience and Mental Health Research Institute, Cardiff University, Cardiff, CF24 4HQ, UK

**Keywords:** visual cortex, size tuning, plasticity, locomotion

## Abstract

Activity of neurons in primary visual cortex is shaped by sensory and behavioral context. However, the long-term stability of the influence of contextual factors in the mature cortex remains poorly understood. To investigate this, we used two-photon calcium imaging to track the influence of surround suppression and locomotion on individual neurons over 14 days. We found that highly active excitatory neurons and parvalbumin-positive (PV+) interneurons exhibited relatively stable modulation by visual context. Similarly, most neurons exhibited a stable yet distinct degree of modulation by locomotion. In contrast, less active excitatory neurons exhibited plasticity in visual context influence, resulting in increased suppression. These findings suggest that the mature visual cortex possesses stable subnetworks of neurons, differentiated by cell type and activity level, which have distinctive and stable interactions with sensory and behavioral contexts, as well as other less active and more labile neurons, which are sensitive to visual experience.

## Highlights

• Highly active excitatory neurons are stably modulated by visual context
• Lower activity neurons exhibit plasticity of influence of visual context in mature V1
• PV interneurons maintain relatively stable modulation by visual and behavioral context
• Majority of excitatory neurons are stably modulated by behavioral context

## Graphical Abstract

**Figure:**
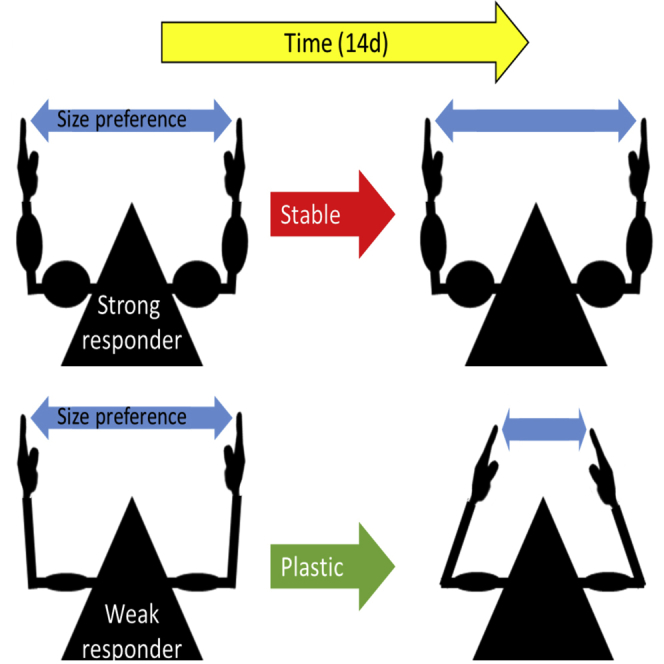

## Teaser

Using chronic in vivo two-photon calcium imaging, Ranson shows that, while subsets of highly active excitatory neurons and PV+ interneurons are stably influenced by contextual factors, less active neurons are subject to experience-dependent plasticity of these influences.

## Introduction

The processing of information by cortical primary sensory circuits is strongly dependent upon both sensory and behavioral contexts. For instance, in the primary visual cortex (V1), stimuli that extend beyond the classical receptive field (RF) of excitatory neurons typically suppress responses to RF stimulation (Adesnik et al., 2012, Blakemore and Tobin, 1972, Gilbert et al., 1996). Conversely, behavioral factors, such as locomotion, modulate the activity of V1 neurons relative to states of quiet wakefulness (Ayaz et al., 2013, Keller et al., 2012, Niell and Stryker, 2010, Saleem et al., 2013).

A number of overlapping circuit elements have been suggested to mediate the effects of sensory and behavioral contexts on visual cortical processing. These include subclasses of local interneurons (Adesnik et al., 2012, Fu et al., 2014, Pi et al., 2013), innervation from the basal forebrain (Fu et al., 2014, Lee et al., 2014), noradrenergic innervation from the locus coeruleus (Polack et al., 2013), and input from the anterior cingulate cortex (Fiser et al., 2016). A fundamental question about these contextual effects, regardless of their origin, concerns the stability versus flexibility of their influence in shaping early sensory processing in the mature cortex. Considerable effort has been invested in exploring the developmental plasticity of a range of rodent V1 receptive field properties during early post-natal development (Gordon and Stryker, 1996, Ranson et al., 2012, Ranson et al., 2013, Ko et al., 2014, Pecka et al., 2014, Wang et al., 2010), as well as the degree of plasticity of “classical” receptive field properties such as orientation tuning and ocular dominance in the adult brain (Andermann et al., 2010, Kerlin et al., 2010, Kreile et al., 2011, Lütcke et al., 2013, Rose et al., 2016). In contrast, many questions remain unaddressed regarding the long-term stability of contextual influences on mature V1 neurons (Gilbert and Li, 2013, Lütcke et al., 2013). Key issues include whether behavioral or visual context modulation is a fixed property of individual V1 neurons or only of the population as a whole; whether the degree of stability of modulation varies between cell types; and whether mature V1 neurons undergo experience-dependent plasticity of contextual influences, as has been previously demonstrated for orientation selectivity and ocular dominance.

We used longitudinal two-photon imaging to track the stability of sensitivity to the sensory and behavioral contexts of putative excitatory pyramidal neurons and parvalbumin-positive (PV+) interneurons over an interval of 14 days, allowing repeated measurements of these features from single neurons. We found that mature V1 exhibits both long-term stability of contextual modulation in a subset of highly responsive neurons and a capacity for significant plasticity of more weakly responding cells, with PV+ inhibitory neurons exhibiting less systematic plasticity in their responses than putative excitatory neurons.

## Results

**Figure 1:**
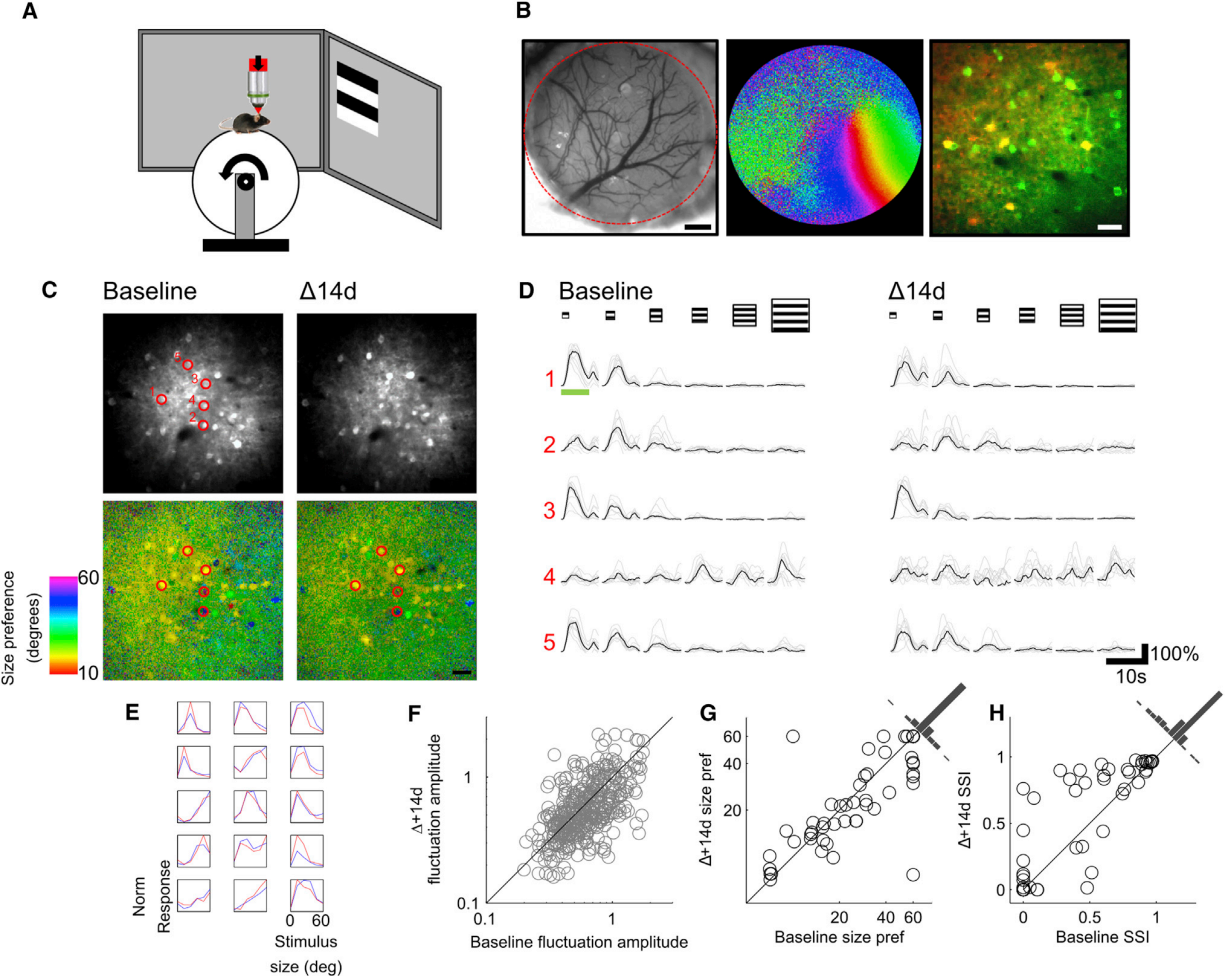
Experimental Setup and Longitudinal Response Stability (A) Schematic of recording setup with mouse on fixed-axis treadmill. (B) View through cranial window, showing cortical vasculature (scale bar, 500 μm); color-coded intrinsic signal imaging map of retinotopy; and co-localization image showing V1 neurons expressing GCaMP6S in all neurons and tdTomato in PV+ cells (scale bar, 30 μm). (C) Representative example of field of view of V1 neurons recorded at baseline (top left) and 14d later (Δ14d; top right). Pixel-wise stimulus preference map from same field of view showing size preference on a pixel-by-pixel basis at baseline and 14 days later (bottom left and right; scale bar, 30 μm). (D) Traces of five example neurons marked in (C) showing responses at baseline and 14d later to size-tuning stimulus, with light traces showing individual trials and black traces showing average response. Green bar shows stimulation period. (E) Example size-tuning curves of 18 highly responsive neurons showing stability of tuning between baseline (blue) and 14d later. Norm, normalized. (F) Between-session correlation in magnitude of calcium signal fluctuations. (G and H) Between-session correlation of size preference (pref.) (G) and surround suppression index (H). See also Movie S1.

In order to measure the stability of contextual factors in modulating neurons in V1, two-photon imaging, together with the genetically encoded calcium indicator GCaMP6S, was used to record longitudinally from putative excitatory and PV+ inhibitory neurons in both stationary alert and running animals (Figures 1A and 1B). This allowed a repeated measurement to be made of the stability of modulation by contextual factors from single neurons (Figures 1C–1E). Expression of the genetically encoded calcium indicator GCaMP6S was driven using an adeno-associated virus (AAV) injected into the primary V1 at a site targeted using intrinsic signal imaging (Figure 1B). In order to assess long-term stability of modulatory effects at the single cell level, neural activity was measured in adult animals (aged postnatal day [P]80–P95) during a baseline session and then again 14 days later (Δ14d). Average population retinotopic preference of recorded neurons was determined using 30° circular drifting gratings before the first session, with subsequent orientation and size-tuning stimuli centered at this preferred retinotopic location.

### Highly Responsive V1 Neurons Exhibit Long-Term Stability of Size Preference over Time

For each field of view, we first calculated pixel-wise maps of size preference in which a high degree of diversity of size preference could be observed (Figure 1C, bottom panels). Single neurons could easily be visually discriminated as clusters of pixels with similar preference, and many of these clusters could be seen to persist over the two sessions. In order to measure size preference of individual cells over the two sessions, regions of interest that correspond to individual neurons were detected using a semi-automated algorithm (see Experimental Procedures), and these were used to extract single-cell responses over the two sessions. Size preference and surround suppression index (SSI; see Experimental Procedures) were measured for putative excitatory neurons that had an orientation preference close to the size-tuning stimulus (±30°, horizontal grating) and exhibited robust visually evoked responses (ΔF/F > 1) that persisted over the two recording sessions. This highly responsive subset of cells was selected to minimize the influence of measurement noise in assessing the stability of surround suppression. Intersession correlation of activity fluctuations in the excitatory population as a whole (quantified as the SD of each neuron’s ΔF/F trace) showed that highly responsive cells tended to be persistently highly responsive (r = 0.57, n = 383, p < 10^−33^; Figures 1F and S1). However, despite this, only 47% of the top 20% most active neurons in the first session were also in this category in the second session, indicating significant motility in average activity levels. Persistently highly responsive cells spanned the full range of size preferences measured (Figures 1D, 1E, and 1G) and exhibited a range of degrees of suppression (Figures 1D, 1E, and 1H). There was, however, a bimodality in the distribution of both size preference and SSI whereby extremes were overrepresented (see Figure S1). Comparison of size-tuning curves between sessions revealed a high degree of intersession correlation of both size preferences (r = 0.80, n = 60, p < 10^−13^; Figure 1G) and SSI (r = 0.86, n = 60, p < 10^−18^; Figure 1H), with a mean absolute difference of 6.9° ± 1.57° in size preferences. While size preference of individual cells did not differ significantly between sessions, there was a net increase in the degree of suppression of 0.08 ± 0.03 (n = 60, p < 0.05). This analysis shows that a subpopulation of persistently highly responsive cells maintain robust size preferences over time, consistent with a scheme of lifetime sparseness (Barth and Poulet, 2012).

### Less Active V1 Neurons Exhibit Robust Experience-Dependent Plasticity of Surround Suppression

**Figure 2:**
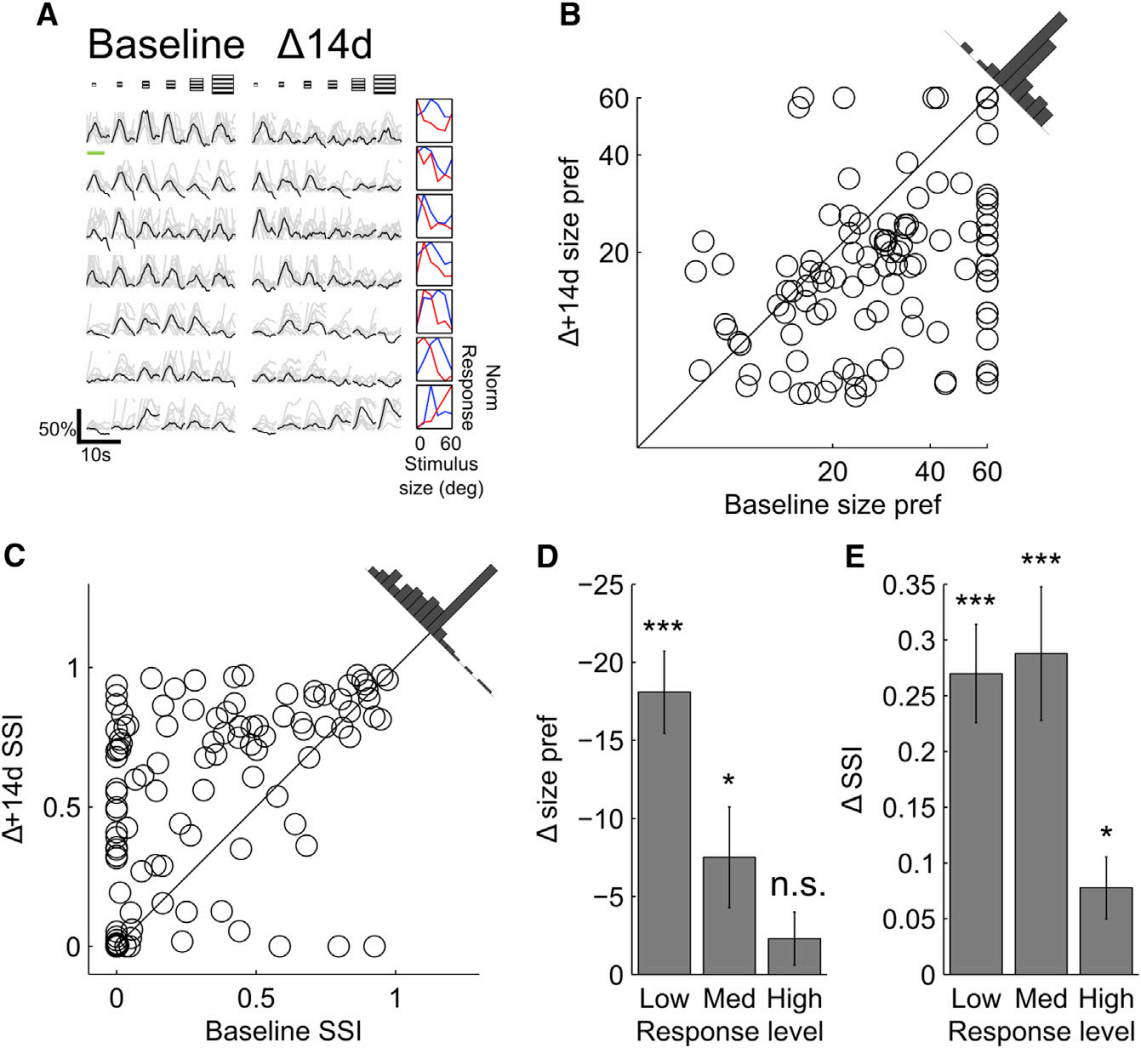
Plasticity of Size Preference and Suppression in Weakly Responding Neurons (A) Traces of seven example neurons marked in showing baseline and Δ14d responses to size-tuning stimulus, with light traces showing individual trials (truncated for display purposes), and black traces showing average response. Green bar shows stimulation period. Normalized (Norm) raw tuning curves are shown on the right. (B and C) Intersession correlation of size preference (pref.) (B) and SSI (C) showing shift in response preferences toward smaller sizes and greater suppression. (D and E) Average net shift in size preference (D) and SSI (E) in low-, medium-, and high-responding neurons shows greater plasticity in weakly responding neurons. All data are presented as mean ± SEM. ^∗^p < 0.05; ^∗∗∗^p < 0.001; n.s., not significant.

The population of excitatory neurons was next divided into three groups, depending on response magnitude to preferred stimuli (ΔF/F: low, 0.3–0.7, n = 48; medium, 0.7–1, n = 19; and high, > 1, n = 60), with a requirement imposed that neurons had to fall into the same category over the two sessions to be included in further analysis. This allowed an examination of whether stability of surround suppression varies depending upon overall responsiveness. While high-responding cells were found to exhibit long-term stability in their degree of modulation by visual context (as described earlier), more weakly responding cells exhibited a higher degree of intersession variability in both size preference and SSI (Figure 2). However, rather than simply reflecting greater measurement noise (e.g., due to lower response magnitudes; see Figure S2 for within- versus between-session analysis) or differences in behavioral state (see Figures S2E and S2F), this intersession variability was, instead, due to a systematic decrease in preferred size and increase in the SSI of the majority of the neurons recorded. In the population of excitatory neurons as a whole, the majority of cells either decreased or maintained preferred size (81%), and increased or maintained SSI (80%), as can be observed in Figures 2B and 2C, in which most data points can be seen to be below and above the unity line, respectively. The degree of net reduction in size preference was next quantified in the three response level groups (Figures 2D and S3), and significant plasticity was observed in both low-responding neurons (size shift = −18.08° ± 2.64°; n = 48, p < 10^−7^, paired t test) and medium-responding neurons (size shift = −7.52° ± 3.23°; n = 19, p < 0.05, paired t test), but not high-responding neurons (size shift = −2.31° ± 1.69°; n = 60, p = 0.18, paired t test), as well as a small but statistically significant reduction in response amplitude in the low-responding group (reduction in ΔF/F = 0.087, p < 0.05; Figure S1E). A similar pattern emerged for SSI (Figure 2E), which was strongly increased in low-responding neurons (SSI shift = 0.27 ± 0.04; n = 48, p < 10^−6^, paired t test) and medium-responding neurons (SSI shift = 0.29 ± 0.06; n = 19, p < 10^−3^, paired t test) and less strongly but significantly increased in strongly responding neurons (SSI shift = 0.08 ± 0.03; n = 60, p < 0.05). These results indicate that, while strongly responding neurons maintain stable size preferences over time and are relatively immune to the effects of visual experience, less active neurons can exhibit a high degree of plasticity, which can be recruited by visual experience, and results in a driving up of suppression and a driving down of preferred stimulus size.

### V1 Neurons Exhibit Longitudinally Stable and Characteristic Modulation of Activity by Locomotion

**Figure 3:**
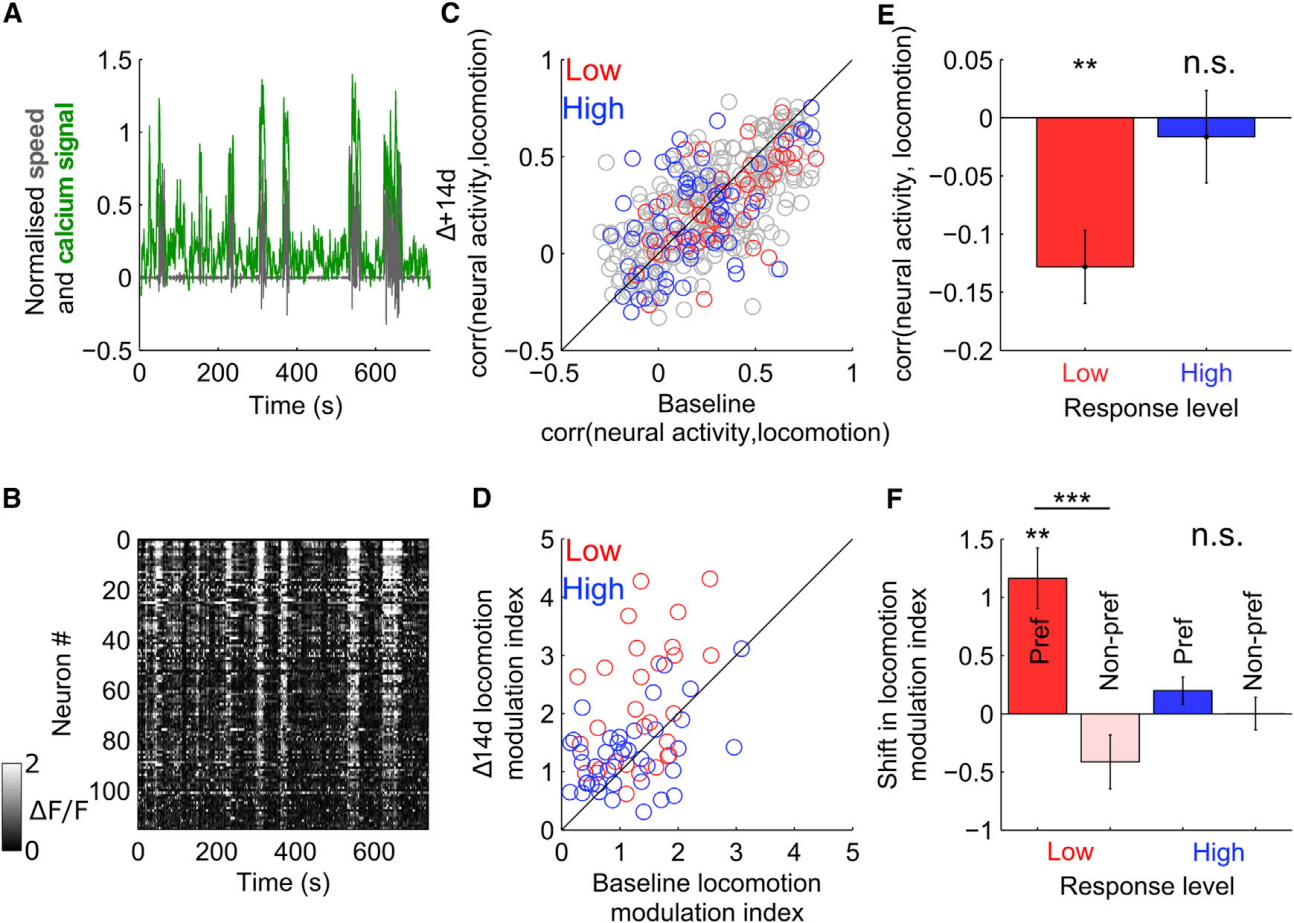
Stability of Modulation of Neural Activity by Locomotion (A) Overlaid normalized traces of locomotion speed (red) and average neural activity of a population of 109 neurons, showing a high degree of correlation between neural activity and locomotion. (B) Activity map of neurons averaged in (A) over the same time period. (C and D) Correlation (corr) of neural activity with locomotion (C) and modulation of visually evoked response to preferred stimulus (D) between baseline session versus session 14d later. (E and F) Between-session shift in corr(neural activity, locomotion) (E) and LMI (F) in low- and high-responding neurons during preferred and non-preferred (Pref and Non-pref, respectively) stimulus presentation. All data are presented as mean ± SEM. ^∗∗^p < 0.01; ^∗∗∗^p < 0.001; n.s., not significant.

We next investigated the stability of the effects of locomotion on visual cortex activity. As has been previously reported, the activity of putative excitatory neurons in V1 was strongly modulated by locomotion (Figures 3A and 3B). The correlation between putative excitatory neuron activity and locomotion was calculated in the population as a whole during ongoing visual stimulation with the size-tuning stimulus. Differences between experiments in proportion of time moving versus stationary introduce variability in locomotion-neural activity correlation. In order to mitigate this problem, data were subsampled to include equal periods of time of locomotion and stationary behavior (see Experimental Procedures for a detailed description and Figure S4 for example cells and summary histograms). The activity of the majority of putative excitatory neurons was correlated with locomotion (Figure 3C), with significant correlation coefficients (p < 0.01, shuffle test) ranging from −0.30 to 0.81, and with a stable fraction of significantly correlated neurons observed over the 14d period (baseline = 88.8%, Δ14d = 89.2%). We next assessed whether the degree of locomotion modulation of individual putative excitatory neurons was stable over the two sessions. Correlating the individual cell’s baseline locomotion correlation with its Δ14d locomotion correlation revealed a strong intersession correspondence across the range of locomotion correlation values (r = 0.64, n = 383, p < 0.01; Figure 3C). This suggests that, despite the proposed relatively indirect mechanisms of modulations of neural activity by locomotion (Fu et al., 2014, Lee et al., 2014), the modulation is, in large part, a stable feature of individual cells over time. In addition, further examination of the low-responding group, which exhibited increases in surround suppression between sessions, revealed that these neurons also exhibited a small but statistically significant reduction in correlation with locomotion (shift = −0.13, p < 0.01, t test, Figure 2E), which was not present in more strongly responding neurons (shift = −0.02, p = 0.68, t test). We next investigated whether the degree of modulation of visually evoked response by locomotion was a stable feature of individual neurons. The degree of locomotion-induced gain of visual responses to preferred size stimuli was quantified with a locomotion modulation index (LMI), defined as (R_M_ − R_S_)/R_S_, where R_M_ and R_S_ are the visual response while moving and while stationary, respectively. LMI was compared between baseline and Δ14d sessions for the subset of neurons that had stationary and locomotion trials for their preferred stimulus size and were either in the low-responding group (ΔF/F, 0.3–0.7) or the high-responding group (ΔF/F > 1). This analysis revealed that, while there was a strong positive correlation between sessions in LMI in the population as a whole (r = 0.48, n = 49, p < 0.01; Figure 3D), there was also a systematic intersession increase in LMI, which was observed in the low-responding group (shift in LMI = 1.04; p < 0.001, paired t test; Figure 3F), but not in the high-responding group (shift in LMI = 0.17; p = 0.15, paired t test; Figure 3F); in addition, this plasticity was limited to responses to the preferred stimulus (Figure 3F). These findings show that the majority of excitatory V1 cells are significantly modulated by locomotion and that, while this modulation varies significantly between neurons, it is, in large part, stable over the 14d interval within single neurons. In addition, these data indicate that, in adult animals, the modulation of visual response by locomotion is plastic.

### PV Interneurons Exhibit a Greater Degree of Long-Term Stability of Modulation by Contextual Factors than Putative Excitatory Neurons

**Figure 4:**
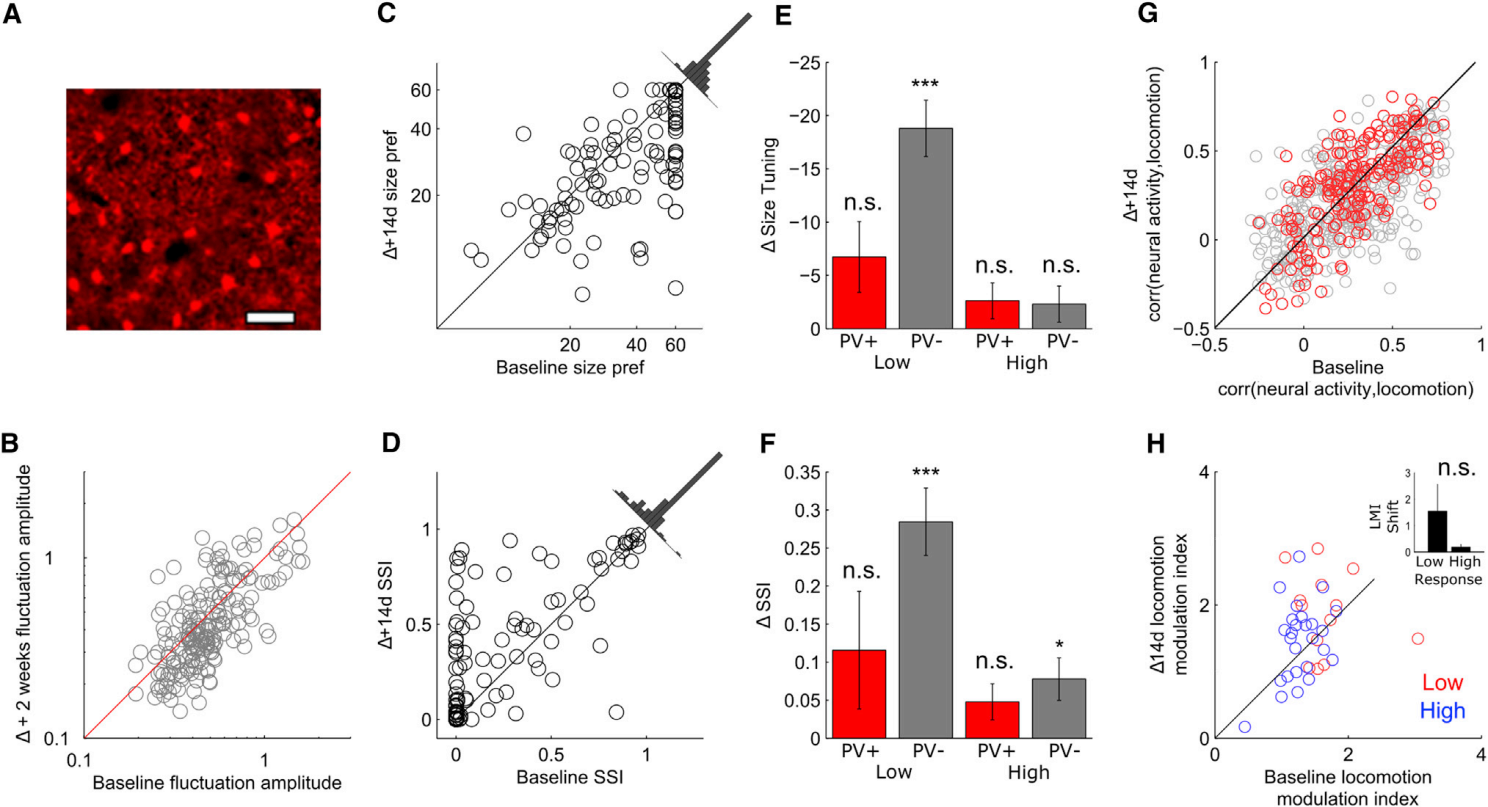
Greater Stability of Size Preference and SSI in PV+ Interneurons (A) Example of average frame of red channel showing labeling of PV+ interneurons expressing tdTomato. (B) Intersession correlation in magnitude of calcium signal fluctuations. (C and D) Intersession correlation of size preference (pref) (C) and SSI (D). (E and F) Comparison of intersession shift in size preference (E) and SSI (F) showing greater intersessional plasticity on average in putative excitatory neurons. (G) Correlation (corr) of neural activity with locomotion between sessions in PV+ interneurons (red) and putative excitatory neurons (gray). (H) Intersession comparison of LMI in low-responding (red) and high-responding (blue) neurons. Inset bar plot shows mean shift in LMI in low- and high-response groups. All data are presented as mean ± SEM. ^∗^p < 0.05; ^∗∗∗^p < 0.001; n.s., not significant.

We next measured the stability of the modulation of PV+ neurons (Figure 4A) by these contextual factors. Previous work has provided evidence that PV+ interneurons receive input from a relatively unbiased sample of local excitatory neurons (Cossell et al., 2015), a model that would predict that PV neurons show relatively homogeneous modulation by contextual factors, with parameters reflecting the average of the excitatory population as a whole (as has been shown with orientation selectivity). Contrary to this prediction, at baseline, PV neurons exhibited heterogeneous size preferences and SSIs and also exhibited a similar bimodal distribution of both size preference and SSI as putative excitatory neurons (Figures S1C and S1D). In common with the putative excitatory population, more active neurons during the baseline session tended to be more active during the Δ14d session (Figure 4B). The intersession correlation of size preference and SSI were next calculated in the PV+ neuron population as a whole (using the inclusion criterion of maintaining a response amplitude across the two sessions of ΔF/F > 0.3) and in the highly responding subset of cells (ΔF/F > 1). Size preferences and SSI were both highly correlated between sessions, both in the population as a whole (size: r = 0.70, n = 123, p < 10^−18^; SSI: r = 0.71, n = 123, p < 10^−20^; Figures 4C and 4D) and in the highly responding subset of cells (size: r = 0.88, n = 36, p < 10^−9^; SSI: r = 0.94, n = 30, p < 10^−13^; Figures 4C and 4D). We next tested for systematic between-session plasticity of size tuning and SSI, as had been observed in putative excitatory neurons, comparing plasticity in weakly and strongly responding cells using the same criteria used in the excitatory population. In common with the putative excitatory population, strongly responding PV+ neurons exhibited no significant shift between sessions in size preference (mean shift = −2.62 ± 1.68; n = 36, p = 0.13; Figure 4E) and, in addition, exhibited no significant shift in SSI (mean shift = 0.05 ± 0.02; n = 36, p = 0.052; Figure 4F). In contrast to the excitatory population, weakly responding PV+ neurons exhibited no significant net shift in either preferred stimulus size (mean shift = −6.74 ± 3.32; n = 19, p = 0.06; Figure 4E) or SSI (mean shift = 0.12 ± 0.08; n = 19, p = 0.15; Figure 4F). A between-cell-type comparison of mean shift in size preference and SSI confirmed a significant difference in PV+ versus parvalbumin-negative (PV−) cells in between-session plasticity (size shift: p < 0.01, t test; SSI shift: p < 0.05, t test). Despite the lack of statistical significance of shifts of size and SSI in PV+ cells, the degree of variability in the weakly responding population, particularly in the shift in SSI, suggests that there may be a subset of PV+ neurons that are more plastic and exhibit a behavior similar to that of weakly responding excitatory neurons but that are not distinguishable by their response amplitude. The stability of the modulation of PV+ neuron activity by locomotion was next measured. As was observed in excitatory neurons, there was both a large fraction of PV+ cells with significant correlations with locomotion (91% in session 1, 95% in session 2; Figure 4G) and a high degree of intersession correlation in degree of modulation (r = 0.71, n = 196, p < 10^−30^). A between-cell-type comparison of baseline neural activity correlation with locomotion revealed no significant difference between the two cell types (PV+, 0.29 ± 0.02; putative excitatory, 0.269 ± 0.01; p = 0.32, t test). We next compared the degree of stability of the modulation of visually evoked responses by locomotion in the low-responding and high-responding PV+ population. In common with the high-responding putative excitatory neurons, high-responding PV+ neurons exhibited no significant mean shift in LMI between sessions (shift = 0.18, n = 36, p = 0.11, paired t test). Low-responding neurons also exhibited no systematic shift that reached statistical significance (shift = 1.55, n = 19, p = 0.15), although, in common with their shift in SSI, there was a high degree of diversity in shift size within this group. These results indicate that the responses of PV+ interneurons are relatively stable in general, compared to the excitatory population, especially in a highly responsive subpopulation, despite significant shifts in the local excitatory population. In particular, the PV+ population did not undergo the robust between-session increase in surround suppression and reduction in preferred stimulus size observed in excitatory neurons, although it did exhibit a weak trend in this direction.

## Discussion

We used chronic two-photon imaging of V1 to make longitudinal measurements of the influence of two types of contextual factors on the activity of visual cortical neurons: surround suppression and modulation of neural activity by locomotion. We found that a subset of highly visually responsive putative excitatory neurons maintained stable size preferences over the 14d period, while, in contrast, less strongly visually responsive neurons were significantly more variable between sessions. Unexpectedly, this greater intersessional variability was not random but was, instead, characterized by a systematic shift toward smaller size preferences and greater surround suppression. This plasticity may be driven by the strong visual stimulation during the baseline experiment (as opposed to normal visual experience between imaging sessions), as the visual cortex is considered to be mature and outside of the developmental critical period at the age studied (Gordon and Stryker, 1996, Wang et al., 2010). In contrast to the putative excitatory population as a whole, the PV+ inhibitory population exhibited a greater degree of stability and did not show strong evidence of plasticity driven by the visual stimulus. Both cell classes showed a range of degrees of modulation of activity by locomotion behavior, but a high degree of between-session stability, so that highly locomotion-modulated neurons in the baseline session continued to be highly locomotion modulated 14d later. In addition, we found evidence, in low-responding neurons in both cell classes, of an increase in LMI between sessions, although this was only found to be robust and statistically significant in putative excitatory neurons.

An outstanding question is in regard to the means by which highly responsive excitatory neurons attain their greater stability. In addition to exhibiting a greater degree of stability, highly responsive excitatory cells in the majority of cases preferred relatively small stimuli and were subject to a high degree of surround suppression. Given that a large fraction of the intersessional variability in more weakly responding neurons appears to be accounted for by systematic stimulus-driven reductions in preferred size and SSI, one explanation could be a ceiling effect whereby highly responsive cells already have as small a size and as high degree of suppression as is achievable. While this may contribute to the greater stability of some cells, this does not appear to be a complete explanation, as there were a large fraction of highly responsive cells stably tuned to intermediate sizes. A second explanation could be that the poorer signal-to-noise ratio of recordings from more weakly responding cells could result in greater intersession variability simply due to greater measurement noise. Again, we believe that this is unlikely to be a dominant factor, as between-session variability was not random in weakly responding cells but was, instead, almost invariably biased toward smaller size preferences and greater SSI. In addition, within-session analysis showed no significant shift in preferred size or SSI (Figure S2).

Recent studies have increasingly established that a previously unexpected degree of plasticity exists in adult sensory cortex (Frenkel et al., 2006, Fu et al., 2015, Sato and Stryker, 2008). The present study further advances our understanding of adult plasticity by demonstrating that surround suppression is a highly plastic receptive field property in adult V1. In the present experiments, plasticity appears to be driven by the high-contrast gratings that are shown to the animal during the baseline recording session, as naturally occurring developmental maturational processes are considered to be complete by this age (Gordon and Stryker, 1996, Pecka et al., 2014, Wang et al., 2010). One intriguing possibility is that suppression is homeostatically regulated such that high levels of activity, as driven by the strong experimental stimulus, may result in upregulation of suppression. This leads to the question of whether the contrary may also be true, that reduced input scales down suppression and, thus, whether similar processes are engaged during other plasticity-inducing paradigms such as monocular deprivation. Indeed, reduction of visual activity by acute dark rearing has previously been shown to enhance visual cortical plasticity, and this may occur, in part, through a reduction of suppressive effects. Another possibility is that the experimental stimulus induces an LTP (long-term potentiation)-like state (Frenkel et al., 2006) at synapses providing suppressive inputs. Either way, these data highlight that, even under “passive” viewing conditions, plasticity occurs in surround suppression, and they prompt the question of the behavioral and perceptual consequences of this.

Previous studies have provided abundant evidence that visual cortical activity is modulated by locomotion with a number of circuit mechanisms proposed, including input from motor cortex (Keller et al., 2012), nicotinic modulated cholinergic input from the basal forebrain (Fu et al., 2014), activation of the mesencephalic locomotor region (Lee et al., 2014), and noradrenergic input from the locus coeruleus (Polack et al., 2013). Further to this, we provide evidence that each neuron has, to a large extent, its own unique degree to which it is locomotion modulated, and this is stably maintained over, at least, the time period studied here. This suggests a precision and stability of this aspect of cortical circuitry that is, perhaps, surprising considering some of the relatively indirect mechanisms by which locomotion has been proposed to modulate V1 activity. It also prompts the question of the functional significance of some neurons maintaining greater degrees of locomotion sensitivity. In addition, we found that the effect of locomotion on visually evoked activity (the gain), is also plastic, specifically in low-responding neurons. The coincidence of the plasticity of surround suppression and locomotion-induced gain provides further evidence of common circuit elements underlying the two operations (Adesnik et al., 2012, Ayaz et al., 2013, Dipoppa et al., 2016).

A final question posed by these results pertains to why the PV+ interneuron population should exhibit reduced levels of intersession plasticity relative to the putative excitatory population of V1 neurons. Current evidence suggests that PV interneurons receive input relatively indiscriminately from local pyramidal neurons, which is thought to account, in part, for their broad orientation tuning (Cossell et al., 2015). We observed that, contrary to a model of broad sampling of the local population’s size preferences, PV+ neurons exhibited a range of size preferences and SSI values. The PV+ population also did not undergo the robust shift in size preference observed in the putative excitatory population as a whole. The latter observation could be for at least two reasons: first, the PV+ cells may be less plastic than the putative excitatory population; second, the PV+ population’s functional input might simply be dominated by the more active and less plastic fraction of the putative excitatory population.

In summary, we showed that a large fraction of visual cortical cells maintain a stable degree of modulation by contextual factors during visual processing over a timescale of weeks and that this stability was particularly apparent among highly responsive putative excitatory and PV+ inhibitory populations of cells. In the case of modulation of activity by locomotion, this indicates that precise and stable circuitry mediate these effects and that there are subsets of neurons that are more closely and persistently involved in integrating locomotion with visual information. Despite this stability, we provide evidence that strong visual stimulation might itself be able to drive long-term increases in suppressive effects and LMI in a more labile and weakly responding subpopulation of excitatory neurons, suggesting a hitherto unexpected degree of plasticity in adult cortex of this receptive field property.

## Experimental Procedures

### Animals and Imaging

All experimental procedures were carried out in accordance with institutional animal welfare guidelines and licensed by the UK Home Office. Chronic awake imaging experiments were carried out in eight adult C57BL/6J background animals with PV interneurons labeled by crossing the B6.Cg-Gt(ROSA)26Sortm14(CAG-tdTomato)Hom/J and B6;129P2-Pvalbtm1(cre)Arbr/J mouse lines (Jackson Laboratory, JAX Stock#007914 and 008069, respectively). Approximately 2 weeks prior to imaging, animals were anesthetized (isoflurance 4% induction, 1.5%–2% maintenance) and implanted with a chronic cranial window and head fixation bar (Goldey et al., 2014), and neurons in V1 (targeted with intrinsic signal imaging) were labeled with GCaMP6S using an AAV with expression driven by the human synapsin promotor. During imaging sessions, animals were placed on a rotary-encoder-coupled cylindrical treadmill, and neurons were repeatedly visualized through the cranial window using a resonant scanning two-photon microscope. Vascular landmarks were used to relocate regions of interest between sessions. Visual stimulation was presented on a calibrated LCD screen using the Psychophysics Toolbox (Brainard, 1997).

### Statistical Methods

Statistical analysis was carried out in MATLAB 2016b using the Statistics toolbox, and group average values are presented throughout as mean ± SEM unless otherwise noted. The statistical significance of comparisons of tuning properties of individual neurons between sessions was determined using a paired-sample two-tailed t test, and all p values < 0.05 were considered significant (^∗^p < 0.05, ^∗∗^p < 0.01, and ^∗∗∗^p < 0.001). Correlations were calculated as Pearson correlation coefficients. Further experimental procedures are available in the Supplemental Experimental Procedures.

## Author Contributions

A.R. designed, conducted, and analyzed experiments and wrote the manuscript.

## Supplemental Information

Document S1. Supplemental Experimental Procedures and Figures S1–S4

Movie S1. Examples of a Plastic and a Stable Neuron from One Imaged Region, Related to Figure 1 Videos of average responses of individual cells in the two sessions.

Document S2. Article plus Supplemental Information
