# Supplementary material for: Stability and Plasticity of Contextual Modulation in the Mouse Visual Cortex
Source: Cell Rep. 2017 Jan 24;18(4):840–8. doi: 10.1016/j.celrep.2016.12.080 (PMC5289925; doi:10.1016/j.celrep.2016.12.080)
Supplement: Document S1. Supplemental Experimental Procedures and Figures S1–S4 [file mmc1.pdf]

Cell Reports, Volume *18*

Supplemental Information

Stability and Plasticity of Contextual Modulation in the Mouse Visual Cortex

Adam Ranson

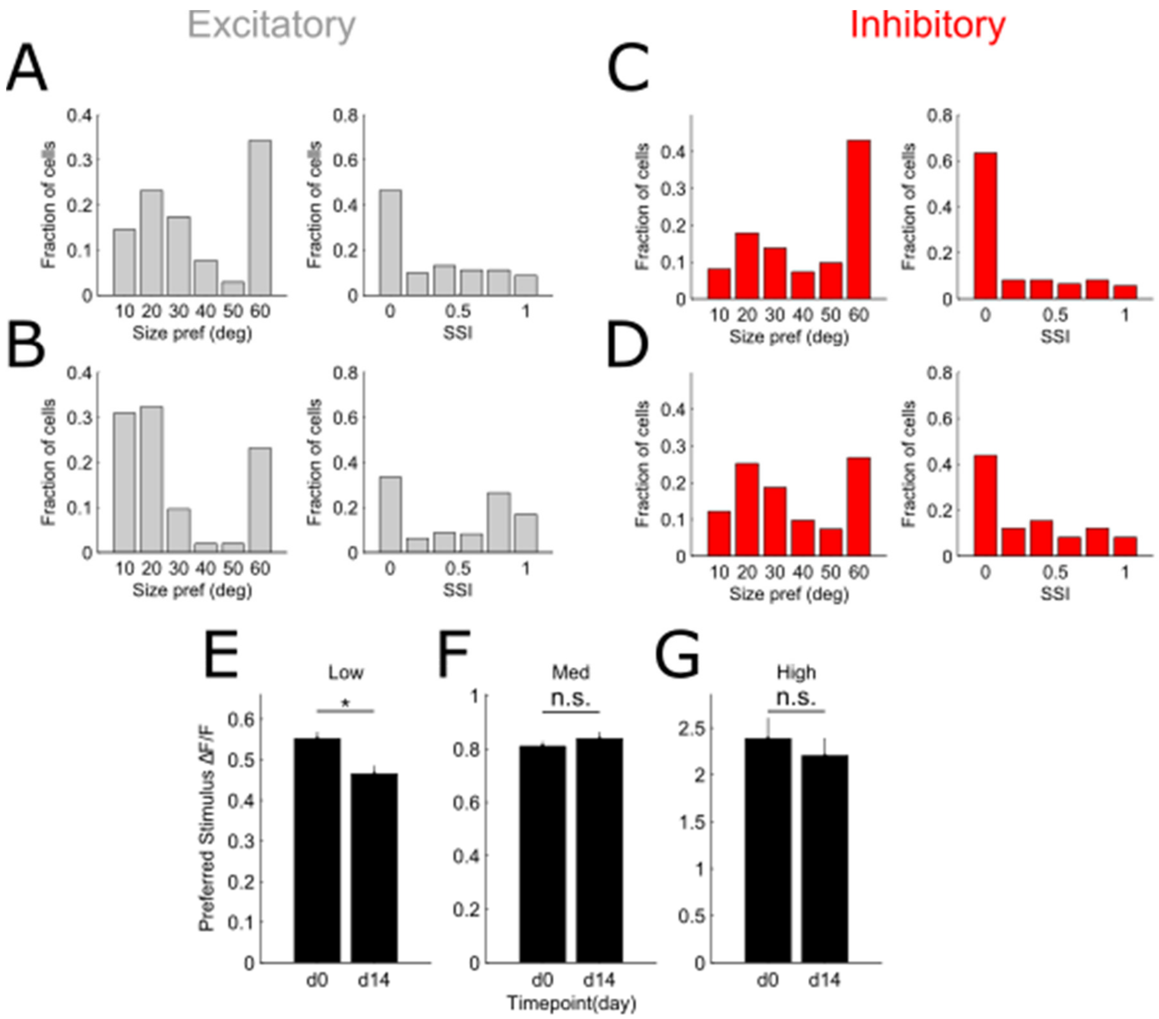

Supplementary Figure 1 **Distribution of size preferences and SSI in putative excitatory and PV+ populations, related to Figure 1.** (A-D) Distribution of size preferences and SSI in putative excitatory neurons at baseline (A), putative excitatory neurons 14d later (B), PV+ neurons at baseline (C) and PV+ neurons 14d later (D). (E-G) Comparison of response amplitude to preferred stimulus at day 0 and day 14 in the 3 response level groups. All data are presented as mean ± SEM $*p < 0.05$; n.s., not significant.

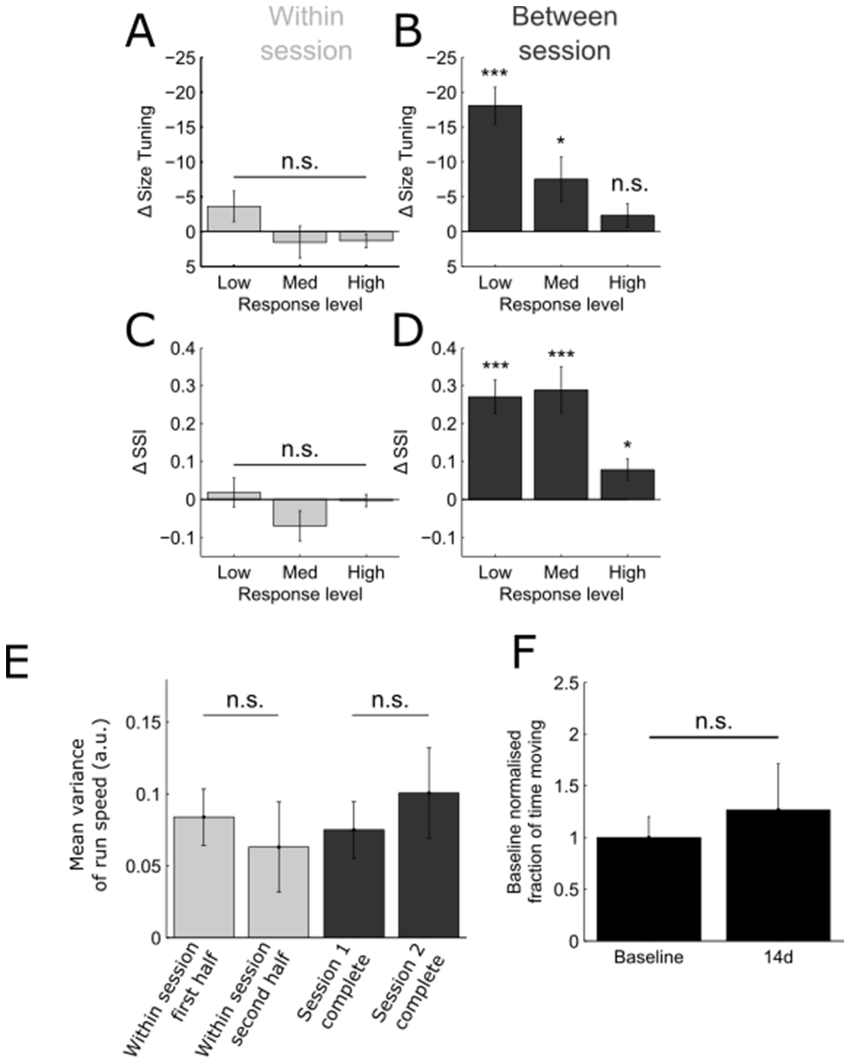

Supplementary Figure 2 **Within session verses between session stability, related to Figure 2.** (A-B) Within session (A) and between session (B) stability of size tuning in the 3 response level groups. (C-D) Within session (C) and between session (D) comparison of SSI in the 3 response level groups. (E) Mean variance in running speed within the first and second half of each session (light grey) and in the first and second session as a whole (dark grey). (F) Normalized fraction of time spent moving in two sessions (n = 8). All data are presented as mean ± SEM ***p < 0.001; **p < 0.01; *p < 0.05; n.s., not significant.

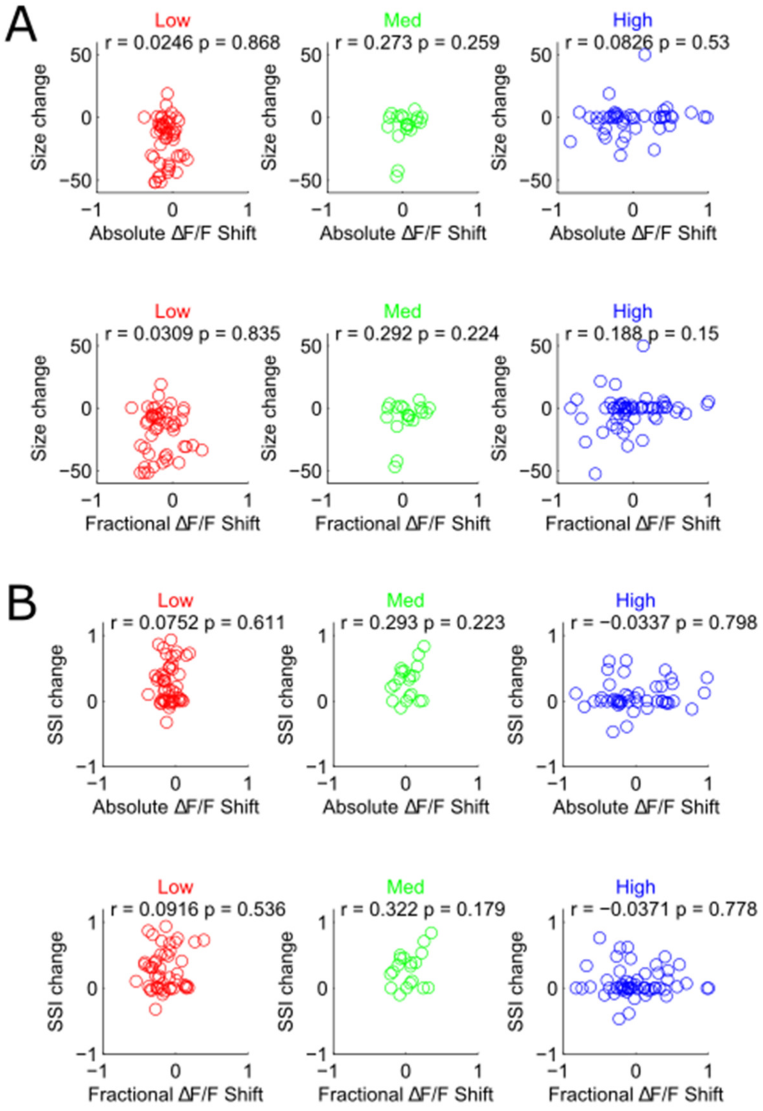

Supplementary Figure 3. **Relationship between shift in response amplitude and shift in size preference and SSI, related to Figure 2.** (A) Correlation between shift in size preference and absolute shift in response amplitude (top row), and relative shift in response amplitude (bottom row), in the 3 response amplitude groups. (B) Correlation between shift in SSI and absolute shift in response amplitude (top row), and relative shift in response amplitude (bottom row), in the 3 response amplitude groups.

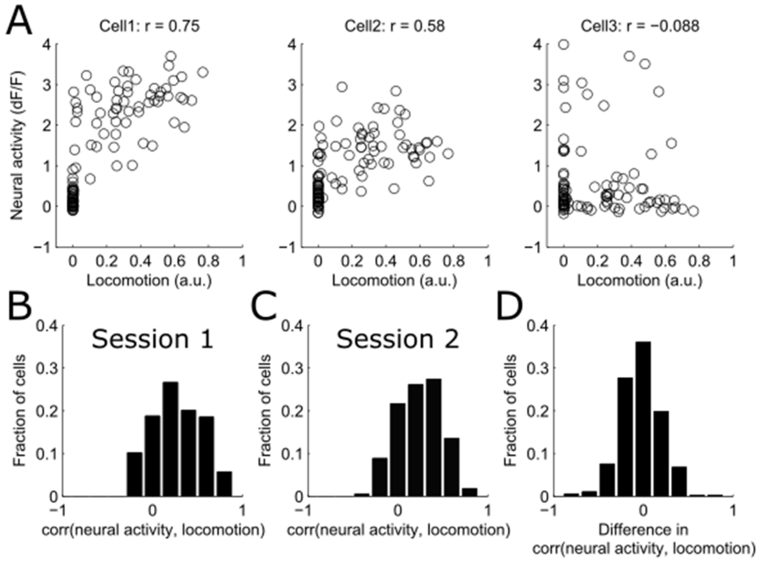

Supplementary Figure 4. **Correlations of neural activity with locomotion, related to Figure 3.** (A) Examples of correlation between neural activity and locomotion in 3 neurons. Data points represent moments in time at which neural activity and locomotion velocity were sampled for individual cells. (B) Distribution of correlation coefficients in session 1 (B) and 2 (C), and distribution of differences in correlation coefficients between sessions (D).

Supplementary Video 1. **Examples of a plastic and stable neuron from one imaged region, related to Figure 1.**

Supplemental procedures

Animals

All experimental procedures were carried out in accordance with institutional animal welfare guidelines, and licensed by the UK Home Office. Experiments were carried out on 8 adult male mice (aged P80-P95) in which PV interneurons were labelled by crossing the B6.Cg-Gt(ROSA)26Sortm14(CAG-tdTomato)Hom/J and B6;129P2-Pvalbtm1(cre)Arbr/J mouse lines (Jackson Laboratory, JAX Stock#007914 and 008069 respectively). Mice were housed under normal light conditions (14h light, 10h dark) and recordings were made during the light period.

Animal surgical preparation and virus injection

Aseptical surgical procedures were conducted based in large part on previously described protocols (Goldey et al., 2014). Approximately one hour prior to cranial window surgery and virus injection, animals were administered with the anti-biotic Baytril (5mg/kg, s.c.) and the anti-inflammatory drugs Carprofen (5mg/kg, s.c.) and Dexamethasone (0.15mg/Kg, i.m.). Anaesthesia was induced and maintained using Isoflurane at concentrations of 4%, and 1.5-2% respectively. After animals were stereotaxically secured, the scalp and periosteum were removed from the dorsal surface of the skull, and a custom head plate was attached to the cranium using dental cement (Super Bond C&B), with an aperture approximately centred over the right primary visual cortex. Transcranial intrinsic signal imaging was then used to determine the precise location of V1, after which a 3mm circular craniotomy was performed, centred on the area of V1 which responded to visual stimulation at an elevation of 20 deg and azimuth of 30-40 deg. Next, 2-3 40nl injections of a virus to drive expression of GCaMP6S (in 5 animals AAV1.Syn.GCaMP6s.WPRE.SV40; titre after dilution $2x10^{11}$ GC/ml; in 3 animals in which only PV neurons were labelled AAV1.Syn.flex.GCaMP6s.WPRE.SV40; titre after dilution $2x10^{11}$) were made into this region at a depth of 200-300μm at sites spaced by approximately 500μm. Injections were made using a microsyringe driver (WPI, UltraMicroPump) coupled to a pulled and bevelled oil filled glass micropipette with a tip outer diameter of approximately 30μm. After injection the craniotomy was closed with a glass insert constructed from 3 layers of circular no 1 thickness glass (1x5mm, 2x3mm diameter) bonded together with optical adhesive (Norland Products; catalogue no. 7106). After surgery animals were allowed at least 2 weeks in which to recover and for GCaMP6S expression to stabilise.

Imaging and locomotor behaviour

In vivo 2-photon imaging was performed using a resonant scanning microscope (Thorlabs, B-Scope) with a 16x 0.8NA objective (Nikon). GCaMP6 and tdTomato were excited at 980nm using a Ti:sapphire laser (Coherent, Chameleon) with a maximum laser power at sample of 50mW. Data was acquired at approximately 60Hz and averaged, resulting in a framerate of approximately 10Hz. Cortical surface vascular landmarks were used to locate the same neurons between sessions. During 2-photon imaging animals were free to run on a custom designed fixed axis cylindrical treadmill, and movement was measured using a rotary encoder (Kübler, 05.2400.1122.0100). Imaging, behavioral and visual stimulation timing data were acquired using custom written DAQ code (Matlab) and a DAQ card (NI PCIe-6323, National Instruments).

In vivo intrinsic signal imaging was performed using previously described methods (Ranson et al., 2013, 2012) using either a custom built system with a MAKO G-125B camera (AVT) or a commercially available system (Imager 3001, Optical Imaging Inc.).

Visual stimuli and experimental design

Mice were first habituated to head fixation in the experimental setup for approximately 15-30 minutes. On the following day, during the first imaging session, the preferred retinotopic location of the field of view of neurons was established using circular 30x30 deg drifting horizontal gratings in a 3x3 grid with temporal frequency of 2 Hz and spatial frequency of 0.05 cycles per degree. Each stimulus appeared and was stationary for 5 seconds, drifted for 2 seconds, was stationary for 2 further seconds and then disappeared. Trials were spaced by 3 seconds, during which a grey screen was displayed. Visual stimuli were generated using the psychophysics toolbox (Brainard, 1997), and displayed on calibrated LCD screens (Iiyama, BT481). Having established retinotopic preference, orientation tuning was next measured using circular gratings with the same temporal and spatial frequency, at the identified preferred location, and displayed at 12 different orientations. Finally size tuning was measured, again using stimuli with the same temporal and spatial frequency, and at the preferred location, with a horizontally orientated grating, with stimuli sizes ranging from 10 – 60 deg in steps of 10 deg. During the second imaging session, 14d later, the same neurons were relocated using vascular landmarks and the size tuning protocol was repeated. It is important to note that while the orientatation, spatial frequency and stimulus centring will inevitably have been suboptimal for many neurons, this was constant between sessions and thus any underestimate of size preference or SSI would have effected both sessions similarly.

Calcium imaging and behavioural data analysis

Brain motion was first corrected for using an automated registration algorithm (Guizar-Sicairos et al., 2008) implemented in Matlab, and data from the second imaging session was registered to the first. A 20µm border was removed from all frames (more than the maximum brain movement observed) to ensure that all pixels were present in all frames in both sessions. Regions of interest were next identified from data acquired during the first session using custom written a semi-automated algorithm based on grouping of pixels with correlated time-courses. Pixels within each region of interest were averaged and background fluorescence contamination was estimated from a 30µm circular area surrounding each soma ROI (excluding other somas) and subtracted from the soma ROI signal with a weighting of 0.7. Only cells with somas which were >5% brighter than surrounding neuropil were included in further analysis. The time series of each ROI was then converted from a raw fluorescence value to ΔF/F with the denominator F value calculated by smoothing the trace and then calculating a sliding window minimum with a window size of approximately 20 seconds. Cells were semi-automatically classified as PV+ based on a thresholded mean registered red channel image, which was eroded and then dilated to remove small areas of labelling of neural processes.

The average responses of neurons to each visual stimulus was calculated, and the max ΔF/F value during the 2s drifting phase was taken as the neuron's response amplitude. Unless the effect of locomotion was being explicitly analysed visual stimulus trials in which animals were moving were excluded. Orientation tuning data were fit using a sum of two Gaussians with identical widths which were constrained such that one peaked at the preferred stimulus, and the peaks were 180 degrees apart (Carandini and Ferster, 2000):

$$R(\Theta) = R_b + R_p e^{-\frac{(\Theta-\Theta_p)^2}{2\sigma^2}} + R_o e^{-\frac{(\Theta-\Theta_p+180)^2}{2\sigma^2}}$$

Here, R(Θ) is the response to the orientation Θ, R_b is the baseline response, R_p is the response to the preferred orientation, R_o is the response to the preferred orientation but with the opposite direction of motion, σ is the tuning width, and brackets indicate orientation values between 0° and 180°.

Size tuning data was fit using a Difference-Of-Gaussians model (DeAngelis et al., 1994):

$$R(s) = R_b + K_e \int_{-s/2}^{s/2} e^{-(2y/a)^2} dy - K_i \int_{-s/2}^{s/2} e^{-(2y/b)^2} dy$$

Here, $R(s)$ is the response to size s, R_b is the baseline response, and the two integrals represent excitatory and inhibitory components. K_e and K_i are the gain of the excitatory and inhibitory components, and a and b represent

space constants. Surround suppression index (SSI) was defined as $(R_{pref}-R_{max})/R_{pref}$ where R_{pref} is the response to the preferred fitted size and R_{max} is the fitted response to the largest size of stimulus. Parameters were fit using the EzyFit Matlab toolbox by a nonlinear minimisation of the sum of squared residuals.

In all experiments animals spent more time stationary than moving. In order to correlate locomotion to neural activity, without introducing artefacts due to differences in fraction of time moving, all locomotion/neural activity samples were selected during which the animal was moving (i.e. m samples, which make up the 'moving set'), and then 100 sets of m locomotion/neural activity samples were randomly selected during which the animal was stationary (resulting in 100 'stationary sets' of m samples). Each of the 'stationary' locomotion/neural activity sets was concatenated with the 'moving' locomotion/neural activity set, resulting in 100 sets of moving locomotion/neural activity data (each with 2*m samples) composed of 50% stationary and 50% moving periods. The corr(neural activity, locomotion) was then calculated for each of the 100 composite sets, and the average correlation coefficient was calculated. In order to compare variation in locomotion within session verses between session (Supplementary Figure 2E), a 10 sec running average was calculated of running speed for each session, mean variance was then calculated of this running average, either in the first and second half of each session, or in the complete first and second session.

Pixel-wise stimulus preference maps were constructed by first calculating the mean of the registered imaging frames recorded during the drifting phase of each stimulus, and then determining for each pixel the stimulus which elicited the largest mean response.

References

Brainard, D.H., 1997. The Psychophysics Toolbox. Spat. Vis. 10, 433–436. doi:10.1163/156856897X00357

Carandini, M., Ferster, D., 2000. Membrane potential and firing rate in cat primary visual cortex. J. Neurosci. 20, 470–484. doi:10.1098/rspb.1986.0060

DeAngelis, G.C., Freeman, R.D., Ohzawa, I., 1994. Length and width tuning of neurons in the cat's primary visual cortex. J. Neurophysiol. 71, 347–374. doi:8158236

Goldey, G.J., Roumis, D.K., Glickfeld, L.L., Kerlin, A.M., Reid, R.C., Bonin, V., Andermann, M.L., 2014. Versatile cranial window strategies for long-term two-photon imaging in awake mice. Nat. Protoc. 9, 2515–2538. doi:10.1038/nprot.2014.165

Guizar-Sicairos, M., Thurman, S.T., Fienup, J.R., 2008. Efficient subpixel image registration algorithms. Opt. Lett. 33, 156–158. doi:10.1364/OL.33.000156

Ranson, A., Cheetham, C.E.J., Fox, K., Sengpiel, F., 2012. Homeostatic plasticity mechanisms are required for juvenile, but not adult, ocular dominance plasticity. Proc. Natl. Acad. Sci. 109, 1311–6. doi:10.1073/pnas.1112204109

Ranson, A., Sengpiel, F., Fox, K., 2013. The role of GluA1 in ocular dominance plasticity in the mouse visual

cortex. J. Neurosci. 33, 15220–5. doi:10.1523/JNEUROSCI.2078-13.2013
