# Supplementary figures and images for: Stability and Plasticity of Contextual Modulation in the Mouse Visual Cortex
Source: Cell Rep. 2017 Jan 24;18(4):840–8. doi: 10.1016/j.celrep.2016.12.080 (PMC5289925; doi:10.1016/j.celrep.2016.12.080)

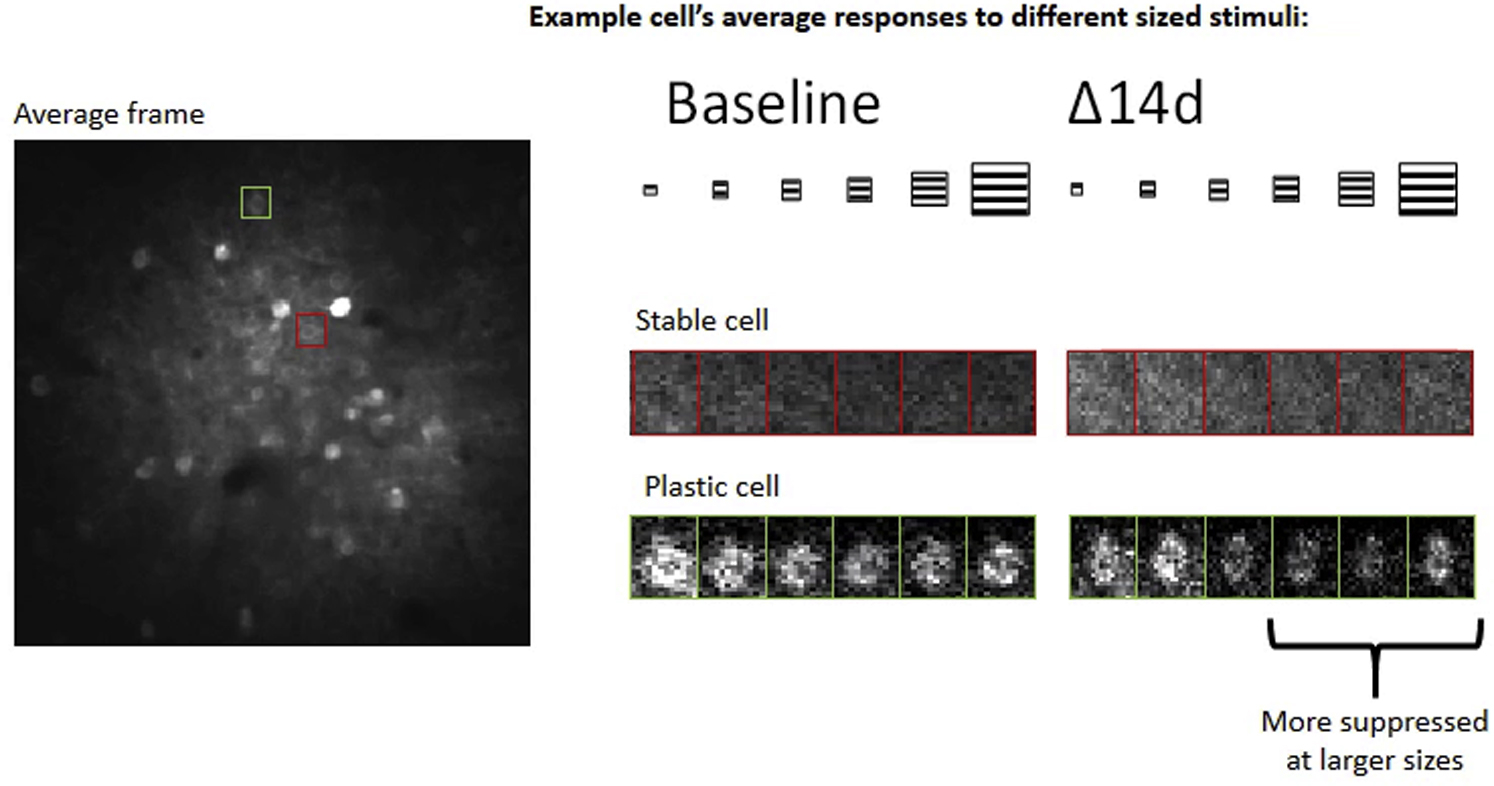

Supplement: Movie S1. Examples of a Plastic and a Stable Neuron from One Imaged Region, Related to Figure 1 — Videos of average responses of individual cells in the two sessions. [file mmc2.jpg]
